# A new measure for in vivo thrombin activity in comparison with in vitro thrombin generation potential in patients with hyper- and hypocoagulability

**DOI:** 10.1007/s10238-016-0417-2

**Published:** 2016-04-19

**Authors:** Oliver Königsbrügge, Silvia Koder, Julia Riedl, Simon Panzer, Ingrid Pabinger, Cihan Ay

**Affiliations:** 1 Clinical Division of Hematology and Hemostaseology, Department of Medicine I, Medical University of Vienna, Waehringer Guertel 18-20, 1090 Vienna, Austria; 2 Department of Blood Group Serology and Transfusion Medicine, Medical University of Vienna, Vienna, Austria; 3 Department of Medicine, Thrombosis and Hemostasis Program, McAllister Heart Institute, University of North Carolina at Chapel Hill, Chapel Hill, NC USA

**Keywords:** Blood coagulation tests, Hemophilia, Thrombin, Thrombophilia

## Abstract

The thrombin generation potential is an in vitro measure for the capacity of an individual to generate thrombin and recognized as a reflection of a hypo- or hypercoagulable status. Measurement of the in vivo thrombin activity, however, may be of clinical significance. We evaluated a new assay for in vivo thrombin activity and compared it to the in vitro thrombin generation potential in patients with hemophilia A (*N* = 15), oral anticoagulation for atrial fibrillation (AF) (*N* = 20), subjects with active cancer (*N* = 21), and healthy volunteers (*N* = 10). Thrombin activity was measured with a commercially available oligonucleotide enzyme capture assay in argatroban-stabilized plasma samples. Thrombin generation potential was determined with a commercially available assay in citrated plasma. Thrombin activity was detected in 17 (30.4 %) patients (mean 0.30 mU/ml [SD 0.80]), and in 39 patients (69.6 %) no thrombin activity was present. In cancer patients, thrombin activity was detected in 11 patients (52 %) (range 0.14–5.00 mU/ml) and was particularly increased in 3 patients with vessel-invasive tumors (1.2, 1.5, and 5.0 mU/ml). In AF patients, thrombin activity was only measureable in two patients (10 %) (recent hematoma [0.4 mU/ml] and recent ischemic stroke [1.5 mU/ml]). Thrombin activity was detected in four patients (27 %) with hemophilia (range 0.29–1.75 mU/ml), all of whom had received a factor VIII infusion on the same day. Thrombin activity did not correlate with any of the parameters of the thrombin generation potential. Only patients in acute procoagulatory states or after clotting factor replacement had elevated in vivo thrombin activity, which was, however, unrelated to the in vitro thrombin generation potential.

## Introduction

Thrombin is a pivotal component of the coagulation cascade, integrally connected in pathways that lead to blood clotting as well as to endogenous anticoagulation and fibrinolysis [1, 2]. The capacity to generate thrombin from circulating prothrombin is considered to reflect an individual’s ability to react to vascular damage and to stop bleeding [3], but low amounts of thrombin are also relevant in preventing thrombosis via the protein C pathway [4]. Thrombin has a short half-life and naturally occurring inhibitors, such as antithrombin, rapidly bind thrombin, which makes the direct measurements of in vivo thrombin difficult [5]. Assays that quantify the thrombin generation potential are in vitro methods that utilize an added coagulation trigger for initiating the formation of thrombin [6]. Elevated and reduced thrombin generation potentials in patients are associated with hyper- and hypocoagulable states, respectively, as well as with thromboembolic outcomes [7–9].

A novel assay for measuring thrombin activity has recently been introduced by Müller et al. [10]. The method is based on an oligonucleotide enzyme capture method that enables the measurement of free thrombin in stabilized plasma samples and is thus independent of an in vitro coagulation trigger. Thus far, measurements of thrombin activity have not been linked to clinical correlates, but very low levels of thrombin activity were measured in plasma from healthy donors compared to subjects with diagnosed thrombophilia [11].

In our current method comparison, we investigated with an exploratory approach how much free circulating thrombin could be measured with the oligonucleotide enzyme capture assay in a selection of clinical cases with hypo- and hypercoagulable states and whether there is a relationship between in vivo thrombin activity and in vitro thrombin generation potential.

## Patients and methods

### Patient samples

Venous blood samples were obtained from patients with confirmed severe hemophilia A, atrial fibrillation (AF), and active cancer in routine care, who supplied written informed consent for clinical surplus samples in accordance with the vote of the local ethics committee. Control samples were obtained from 10 healthy volunteers. Blood was sampled from cubital venipuncture using a 21-gauge needle, after discarding the first 8 ml of blood, and collected without stasis into a citrate plasma vial (Vacuette, Greiner-Bio One, Kremsmünster, Austria; containing 1/10 volume sodium citrate at 0.129 mmol/l) and into an argatroban-containing vial (Thrombin blood collection tubes, Sekisui Diagnostic, Pfungstadt, Germany). The reversible formation of argatroban–thrombin complexes inhibits the degradation of free thrombin in plasma. Vials were centrifuged at 2500 g for 15 min at room temperature, divided into aliquots, and stored at −80 °C within 1 h of blood draw. Prothrombin time (PT, Thromborel S, Siemens Healthcare Diagnostics Products GmbH, Marburg, Germany), activated partial thromboplastin time (aPTT, Pathromtin SL, Siemens), diluted thrombin time (dTT, Hemoclot, Hyphen Biomed, Neuville–sur–Oise, France), and international normalized ratio (INR) were measured with commercially available kits on an accredited process with fresh blood samples.

### Thrombin activity assay

Sample aliquots were thawed in a water bath for 15 min at 37 °C. Thrombin activity was measured using a commercially available fluorogenic assay (OLIGOBIND^®^ Thrombin activity, Sekisui Diagnostic, Pfungstadt, Germany) according to manufacturer instructions. Standards and patient samples were pipetted in duplicate into separate wells of the aptamer-coated microtiter plate and incubated at room temperature for 1 h in the dark, before washing four times with wash buffer. Addition of a thrombin substrate and a fluorogenic substrate to the aptamer-bound thrombin started the cleavage of the fluorogenic substrate. We measured the increase in fluorescence at room temperature with extinction at 360 nm and emission at 460 nm over 30 min at 5-min intervals inside a fully automated, computer-controlled microplate reader (BioTek^®^, FLX800). The rate of change in fluorescence is calculated to thrombin activity (mU/ml) using a 6-point standard curve. Manufacturer-specified limit of detection is 0.10 mU/ml thrombin, and the lower limit of quantification is 0.35 mU/ml thrombin. We found a residual standard error of 11.6 % for the third calibration point at 0.8 mU/ml after 8 repeated measurements of the calibrators.

### Thrombin generation assay

Thrombin generation was measured using a commercially available fluorogenic assay (Technothrombin TGA, Technoclone, Vienna, Austria) according to manufacturer instructions and as described previously [12]. Coagulation of the thawed platelet-poor plasma samples was initiated with addition of the TGA RC low reagent, containing a final concentration of 5 pM recombinant human tissue factor lipidated in 3.2 µmol/l phospholipid micelles (phosphatidylcholine [2.56 µmol/l] and phosphatidylserine [0.64 µmol/l]). The generated thrombin cleaves the fluorogenic substrate Z-Gly-Gly-Arg-AMC (1 mM, + 15 mM CaCl_2_) (Technoclone), while fluorescence was measured at 360 nm extinction and 460 nm emission on the same microplate reader over a period of 120 min in 1-min intervals. The parameters lag time, peak thrombin, time to peak thrombin, velocity index [VI], and area under the curve were used in the analysis. Prothrombin fragment 1 + 2 [F1 + 2] levels were measured as an indirect measure of thrombin with an enzyme-linked immunoassay (Enzygnost F1 + 2; Dade-Behring, Marburg, Germany) according to manufacturer instructions.

### Statistical methods

For the description of the study cohorts, we give continuous variables of patient characteristics by mean and standard deviation (SD) and dichotomous variables by absolute and relative frequency. We compared the parameters of thrombin generation potential (peak thrombin, lag time, time to peak, area under the curve as an expression of the endogenous thrombin potential [ETP] and velocity index [VI]) and F1 + 2 levels with the thrombin activity using the Pearson correlation coefficient, as well as visually using a scatter plot of thrombin activity versus peak thrombin generation potential. Mean comparisons between the subgroups of patients and the healthy controls were calculated with the Student’s *t* test, and a *p* value smaller than 0.05 was considered statistically significant. All calculations were performed with SPSS (version 23, IBM, USA).

## Results and Discussion

Clinical surplus samples were collected from 56 patients, 19 women and 37 men (21 cancer, 20 AF, and 15 severe hemophilia A patients) with a mean age of 54.9 years (cancer patients 59.6, AF patients 69.0 years, and hemophilia patients 29.5 years). Entities of cancer were six lung, five pancreas, three breast, two stomach, and five other cancers.

Hemophilia A patients had a mean baseline factor VIII activity of 0.87 % (SD 1.4), and at blood sampling, a mean FVIII activity of 8.4 % (SD 11.2). At the time of blood collection, four patients with severe hemophilia (defined as <1 % baseline factor VIII activity) had had prophylactic FVIII replacement before blood collection and their mean FVIII activity was significantly higher than the mean FVIII activity of patients in their washout phase since the last factor replacement (21.0 vs. 1.9 %, *p* = 0.002). AF patients had a mean CHA_2_DS_2_-Vasc score of 4.1 (SD 1.4). All AF patients were on continuous anticoagulation treatment at the time of sampling and within the on-treatment range, 4 on dabigatran (mean aPTT 56.6 s, STD 24.12; mean dabigatran concentration [by dTT] = 127.5 ng/ml, STD 195.0), 2 on apixaban (mean PT = 79.0 %, STD 4.24), 9 on rivaroxaban (mean PT = 67.6 %, STD 23.5), and 5 on the vitamin K antagonist phenprocoumon (mean INR = 1.88, STD 0.39).

The oligonucleotide enzyme capture assay detected levels of thrombin activity in 17 patients (30.4 %) with a mean of 0.30 mU/ml (SD 0.80), while no thrombin activity at all was detected in 32 patients (69.6 %) and in all of the healthy controls. In a previous investigation, with plasma from healthy donors, the thrombin activity had been below the assay’s lower limit of quantification and even limit of detection [10]. Therefore, we were able to confirm with our findings that in patients without ongoing coagulation there is little to no free thrombin circulating. In part, this is due to the very short half-life of thrombin, which is degraded quickly in plasma [11]. Nonzero thrombin activity was evenly distributed among men and women (31.8 and 31.6 %, without hemophilia patients), and mean age was not significantly lower (56.5 years [SD 18.5] vs. 51.1 years [SD 18.0], *t* test *p* = 0.32).

**Fig. 1:**
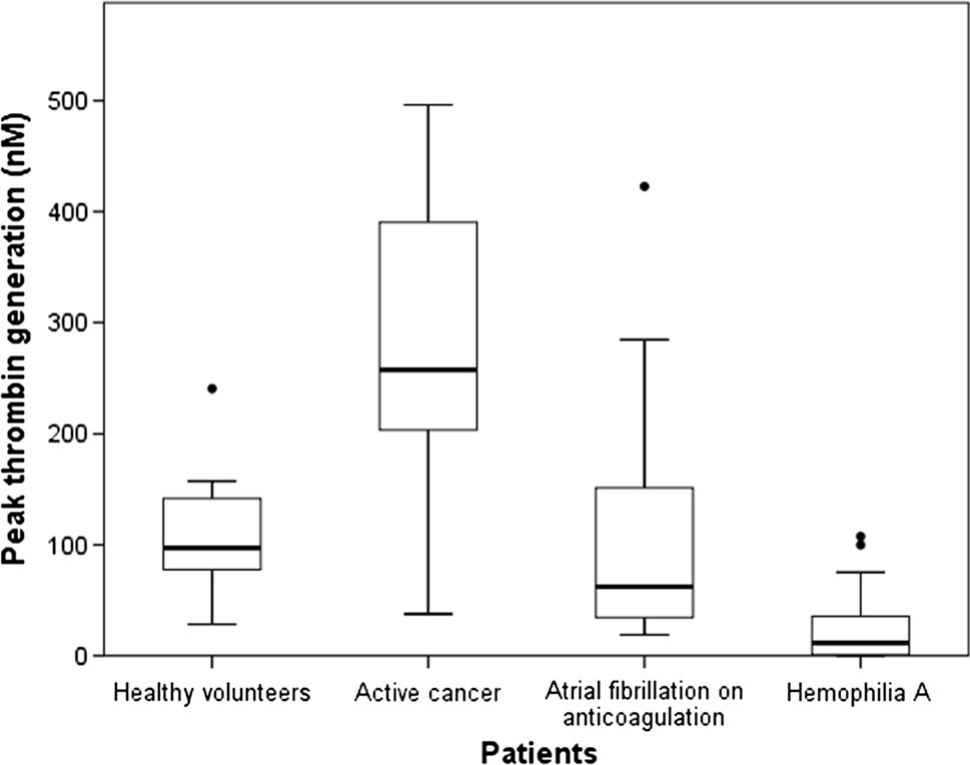
*Box plots* of peak thrombin generation potential. The *thick central line* *inside the* *boxes* represents the median peak thrombin generation, and the boundaries of the box show the 25th and 75th percentile of the data. The *whiskers* are drawn up to the highest and lowest value within 1.5 times the box length. Extremes are drawn with *dots* beyond 1.5 times the box length

Overall patients and healthy volunteers, the mean peak thrombin generation potential was 144.1 nM thrombin (SD 133.6 nM), with a mean lag time of 24.6 min (13.7 min), a mean peak time of 33.6 min (20.4 min), a mean VI of 24.6 nM/min (33.4 nM/min), and mean ETP of 2208.1 nM thrombin (1249.0 nM). The thrombin generation potential parameters are shown separately for patient subgroups in Table 1. Corresponding to previous findings, the parameters of thrombin generation expressed a procoagulatory state in cancer patients (Table 1), which has been associated with an elevated risk for occurrence of venous thromboembolic events [13]. The mean F1 + 2 level was 165.0 pmol/l (SD 110.3) and F1 + 2 levels correlated with all parameters of the thrombin generation over the entire cohort (data not shown). Hemophilia A patients had decreased parameters of thrombin generation potential compared to healthy volunteers and the other patient groups, indicating a reduced capacity for generating thrombin in the event of bleeding, which has also been associated with the severity of hemophilia as well as the severity of the bleeding phenotype in hemophilia patients [14, 15]. The means of the thrombin generation parameters and F1 + 2 levels in patients with AF were statistically close to those of healthy volunteers, except for a significantly longer lag phase and peak time (Table 1). These findings indicate that within our selection of patients the thrombin generation potential and the F1 + 2 levels consistently show a trend from a hypocoagulable state in severe hemophilia A patients to a state of hypercoagulability in cancer patients (Fig. 1).

**Table 1:** Thrombin generation and thrombin activity measurements in separate subgroups of patients

	Healthy volunteers (*N* = 10)	Cancer patients (*N* = 21)	AF patients (*N* = 20)	Hemophilia patients A (*N* = 15)	Cancer patients versus healthy volunteers *t* test *p* value	AF patients versus healthy volunteers *t* test *p* value	Hemophilia A patients versus healthy volunteers *t* test *p* value	Pearson r with thrombin activity (*p* value) (*N* = 17)^a^
TG lag time (min), mean (SD)	20.2 (3.9)	13.27 (4.94)	28.2 (9.5)	38.57 (16.49)	<0.001	0.003	0.001	−0.292 (0.255)
TG peak thrombin (nM), mean (SD)	112.0 (58.7)	272.79 (124.61)	112.4 (107.1)	27.65 (37.21)	<0.001	0.987	0.001	0.361 (0.155)
TG peak time (min), mean (SD)	33.1 (6.1)	19.05 (6.95)	43.5 (17.7)	41.43 (29.84)	<0.001	0.025	0.307	−0.263 (0.309)
TG VI (nM/min), mean (SD)	9.8 (6.7)	58.13 (39.47)	13.8 (16.1)	1.76 (2.73)	<0.001	0.340	0.004	0.304 (0.235)
TG ETP (nM), mean (SD)	2720.4 (924.2)	2808.28 (660.31)	2277.0 (1284.6)	934.25 (1165.15)	0.791	0.290	<0.001	0.323 (0.206)
Prothrombin fragment 1 + 2 (pmol/l), mean (SD)	110.4 (50.5)	282.1 (103.9)	123 (76.9)	93.5 (25.9)	<0.001	0.645	0.280	−0.051 (0.847)
Thrombin activity (mU/ml), mean (SD)	0.00	0.47 (1.11)	0.10 (0.34)	0.20 (0.46)	0.065	0.230	0.115	1

The correlation between parameters of the thrombin generation and thrombin activity is shown by the Pearson correlation coefficient, and means of the measurements are tested for equality by *t* test

*AF* atrial fibrillation, *TG* thrombin generation, *VI* velocity index, *ETP* endogenous thrombin potential

^a^Only patients with nonzero thrombin activity

**Fig. 2:**
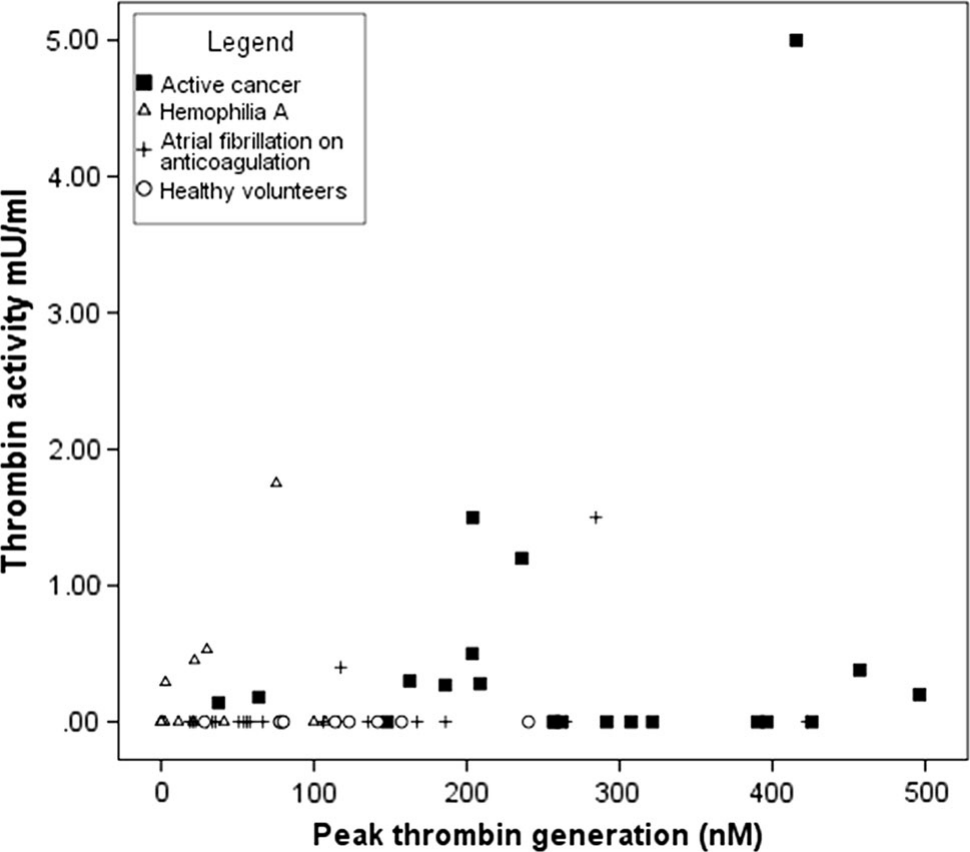
*Scatter plot* of thrombin activity versus peak thrombin generation potential

However, we saw no correlation between all nonzero thrombin activity measurements with the parameters of the thrombin generation or the F1 + 2 levels (Table 1; Fig. 2). In the diverse selection of patients in this investigation, a linear relationship between thrombin activity and thrombin generation potential is not possible, because of the integral connection of thrombin in pathways for clotting, endogenous anticoagulation, and fibrinolysis. The thrombin activity, which is below the limit of detection of the assay, may have substantial effects on preventing wrongful coagulation [4]. Also, an inverse relationship between thrombin activity and thrombin generation could have been possible. We could explain it with a coagulation process that consumes the thrombin generation potential, indicated by a high thrombin activity. A separate analysis within the patient subgroups was not possible due to the small number of patient samples with nonzero thrombin activity. In cancer patients, thrombin activity was detected in 11 (52 %) patients (range 0.14–5.00 mU/ml) and was particularly increased in 3 patients with vessel-invasive tumors (1.2, 1.5 and 5.0 mU/ml). In AF patients, thrombin activity was only measureable in two patients (10 %): One patient had peroneal palsy due to an acute hematoma (0.4 mU/ml), and the other patient had a recent history of ischemic stroke (1.5 mU/ml). Whether the thrombin activity also correlates with the expected increase in thrombin generation potential in patients with acute ischemic stroke [16] is uncertain without further data from samples from a larger cohort with acute thromboembolic events. While differences in thrombin generation potential on treatment with different anticoagulants and at different concentrations were described in previous studies [17–19], there is no evidence for any effect of anticoagulant use or drug plasma levels on thrombin activity levels from our data. The hemophilia patients who had not received a factor VIII replacement preceding the blood sampling had thrombin activity levels beneath the level of detection and significant lower FVIII levels (mean 1.9 (SD 2.3) vs. 21.0 (SD 11.3), *t* test *p* = 0.002). Interestingly, thrombin activity was detected in four patients (27 %) with hemophilia A (range 0.29–1.75 mU/ml), all of whom had received a prophylactic factor VIII infusion on the same day and had higher median FVIII activity. This is a novel finding describing a response in thrombin activity from hemophilia patient plasma after factor VIII replacement. The clinical application of a thrombin activity assay may generate a novel quality to measuring the activity of coagulation factor replacement therapy in hemophilia patients or an alternative for the monitoring of bypassing agent therapy in hemophilia patients with inhibitors [20], if the findings can be reproduced in a larger cohort. Although unlikely, we can presently not exclude that the factor concentrates contained small amounts of thrombin.

The current study is a pilot study to investigate the potential implications of the thrombin activity assay for clinical practice in different cohorts of patients. To our knowledge, there are currently no published studies investigating this new enzyme capture assay for thrombin activity in any clinical setting. We therefore chose a heterogeneous group of patients in order to get an impression of the potential clinical and scientific implications of the assay’s results. The small number of patients in this investigation is a limitation, because only 30 % of patients had thrombin activity levels above the limit of detection and we were unable to perform more analyses within the groups of patients. However, we showed that in most patients with a hyper- or hypocoagulable status no free thrombin is detectable with this assay. Unlike the thrombin generation potential, thrombin activity therefore does not stand for the presence of a state of coagulability but for a depiction of an instantaneous level of thrombin in plasma. This is indication for the occurrence of subclinical coagulation, the complete clinical or experimental implications of which are yet to be elucidated. We saw, however, elevated levels of thrombin in patients with an acute procoagulatory stimulus, which describes a new quality for thrombin measurements. Whether the elevated levels of in vivo thrombin activity may be reproducible for all patients with acute procoagulatory states needs to be confirmed in larger cohorts of specifically selected patients.
